# The complete chloroplast genome sequence of *Oxytropis bicolor* Bunge (Fabaceae)

**DOI:** 10.1080/23802359.2019.1682479

**Published:** 2019-10-24

**Authors:** Chun Su, Pei-Liang Liu, Zhao-Yang Chang, Jun Wen

**Affiliations:** a College of Life Sciences, Northwest A&F University, Yangling, Shaanxi, People’s Republic of China;; b Department of Botany, National Museum of Natural History, Smithsonian Institution, Washington, DC, USA;; c College of Life Sciences, Northwest University, Xi’an, Shaanxi, People’s Republic of China

**Keywords:** Chloroplast genome, *Oxytropis*, intron loss, Fabaceae

## Abstract

The first complete chloroplast genome of *Oxytropis bicolor* Bunge is reported and characterized in this study. The whole chloroplast genome was 122,461 base pairs in length with 110 genes, including 76 protein-coding genes, 30 tRNAs, and 4 rRNAs. In addition, the *atpF* intron was absent. Maximum-likelihood (ML) phylogenetic analysis indicated that *O. bicolor* and species of *Astragalus* were closely related, which is congruent with previous studies.

The genus *Oxytropis* DC. (Fabaceae) comprises 330 species in 6 subgenera and 25 sections (Welsh 2001). Some species of *Oxytropis* are known as locoweeds, which are toxic to many animals. *Oxytropis* is considered closely related to the largest flowering plant genus *Astragalus* L. (Shavvon et al. 2017). *Oxytropis* belongs to the inverted-repeat-lacking clade (IRLC) of Fabaceae. The IRLC is characterized by the lacking of a ca. 25k base pairs (bp) inverted repeat region of the chloroplast genome (Wojciechowski et al., 2004). Morphologically *Oxytropis* can be distinguished easily by the presence of a beak at the tip of the keel petal. *Oxytropis bicolor* Bunge is distributed widely in the arid and semi-arid regions of China and Mongolia.

The DNA sample of *O. bicolor* was extracted from silica gel-dried leaves. A voucher specimen was collected from Shaanxi Province, China (36°55′55.1″N, 110°22′16″E) and stored in the Herbarium of Northwest University (WNU) (collection number: *P. L. Liu 2018166*). The total genomic DNA was extracted by the SDS method (Dellaporta et al. 1983). The genomic library with an insert size of 500 bp was prepared using a NEBNext^®^ Ultra™ DNA Library Prep Kit from Illumina. It was sequenced on an Illumina Hi-Seq 2500 platform (Illumina, San Diego, CA). Raw data were filtered by the program Trimmomatic v.0.33 (Bolger et al. 2014). The filtered reads were used to assemble the chloroplast genome using the programme NOVOPlasty (Dierckxsens et al. 2017) with the *rbcL* gene sequence as the seed (GenBank accession number KU666554). The assembled chloroplast genome was annotated by PGA (Qu et al. 2019) and Geneious prime 2019 (https://www.geneious.com), followed by manual adjustments. The annotated complete chloroplast genome was submitted to GenBank with accession number MN255323.

The complete chloroplast genome of *O. bicolor* was 122,461 bp in length with a GC content of 34.2%. The chloroplast genome of *O. bicolor* has only one inverted repeat (IR) region. The new sequence possessed a total of 110 genes, including 76 protein-coding genes, 4 rRNA genes, and 30 tRNA genes. Usually, the *atpF* gene has a conserved group II intron (Daniell et al. 2008). However, the *atpF* intron is absent in *O. bicolor.* The *atpF* gene of *O. bicolor* is 558 bp long. While the *atpF* gene of *Astragalus mongholicus* Bunge (accession number KU666554) is 1256 bp long, including one intron of 677 bp, exon 1 of 168 bp, and exon 2 of 411 bp. The *atpF* intron loss is rare in flowering plants and has been reported in *Manihot* of Euphorbiaceae (Daniell et al. 2008) and in *Passiflora* of Passifloraceae (Jansen et al. 2007).

To understand the phylogenetic relationship of the newly sequenced *O. bicolor* with other related genera, a maximum-likelihood tree was constructed using the program IQ-TREE (Nguyen et al. 2015) in GTR model with 17 published chloroplast genomes of Fabaceae retrieved from GenBank. All the chloroplast genome sequences were aligned by MAFFT v.7 (Katoh and Standley 2013). GenBank accession numbers were given in Figure 1. Our phylogenetic analysis shows that *Oxytropis* is sister to *Astragalus* (Figure 1), which is congruent with previous studies (Shavvon et al. 2017).

**Figure 1.**
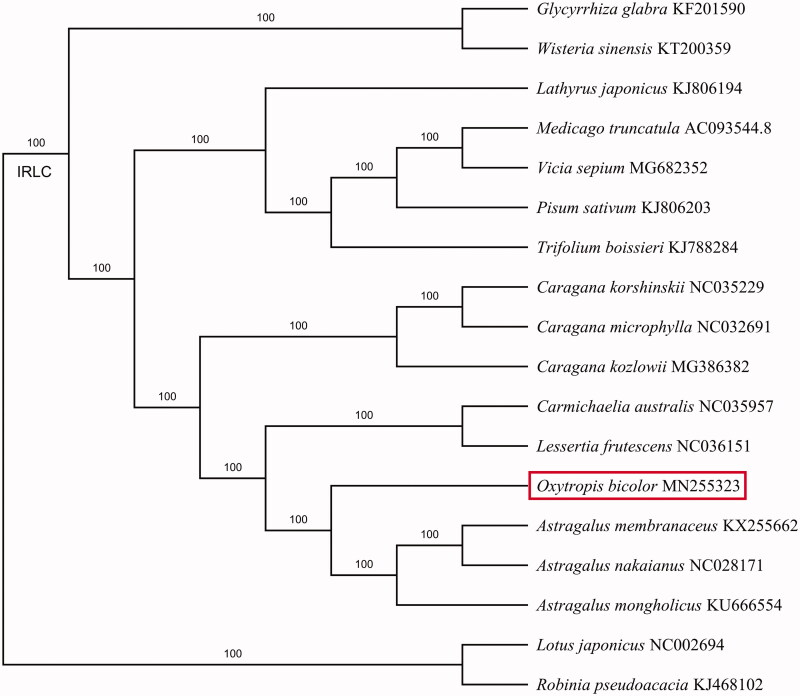
Maximum-likelihood phylogenetic tree inferred from 18 chloroplast genomes of Fabaceae. The position of *Oxytropis bicolor* is shown in a red box. The bootstrap values based on 1000 replicates are shown above each node.
